# Various Organ Damages in Rats with Fetal Growth Restriction and Their Slight Attenuation by *Bifidobacterium breve* Supplementation

**DOI:** 10.3390/life13102005

**Published:** 2023-10-02

**Authors:** Masahiro Tsuji, Nao Tanaka, Hitomi Koike, Yoshiaki Sato, Yoshie Shimoyama, Ayaka Itoh

**Affiliations:** 1 Department of Food and Nutrition, Kyoto Women’s University, Kyoto 605-8501, Japan; 2 Division of Neonatology, Center for Maternal-Neonatal Care, Nagoya University Hospital, Nagoya 466-8560, Japan; yoshiaki@med.nagoya-u.ac.jp; 3 Department of Pathology, Nagoya University Hospital, Nagoya 466-8560, Japan

**Keywords:** probiotics, *Bifidobacterium breve*, fetal growth restriction, low birthweight, kidneys, small intestine, skeletal muscles

## Abstract

Children with fetal growth restriction (FGR) and its resultant low birthweight (LBW) are at a higher risk of developing various health problems later in life, including renal diseases, metabolic syndrome, and sarcopenia. The mechanism through which LBW caused by intrauterine hypoperfusion leads to these health problems has not been properly investigated. Oral supplementation with probiotics is expected to reduce these risks in children. In the present study, rat pups born with FGR-LBW after mild intrauterine hypoperfusion were supplemented with either Bifidobacterium breve (*B. breve*) or a vehicle from postnatal day 1 (P1) to P21. Splanchnic organs and skeletal muscles were evaluated at six weeks of age. Compared with the sham group, the LBW-vehicle group presented significant changes as follows: overgrowth from infancy to childhood; lighter weight of the liver, kidneys, and gastrocnemius and plantaris muscles; reduced height of villi in the ileum; and increased depth of crypts in the jejunum. Some of these changes were milder in the LBW-B.breve group. In conclusion, this rat model could be useful for investigating the mechanisms of how FGR-LBW leads to future health problems and for developing interventions for these problems. Supplementation with *B. breve* in early life may modestly attenuate these problems.

## 1. Introduction

Epidemiological studies have shown that the intrauterine environment is crucial for the future health of each individual, i.e., a concept of DOHaD (developmental origins of health and disease) [1]. Such influence has been particularly shown for cardiovascular diseases, obesity, type 2 diabetes, renal diseases, metabolic syndrome, and sarcopenia [1,2,3]. The most common and typical phenotype of fetuses suffering from an adverse intrauterine environment is fetal growth restriction (FGR), also known as intrauterine growth restriction, and its resultant low birthweight (LBW). As clinical studies on pregnant women and infants born with LBW due to FGR are challenging to conduct, studies on LBW-FGR animal models are indispensable for investigating the influence of LBW-FGR. In developed countries, more than half of the cases of FGR in normally formed fetuses are reportedly caused by placental insufficiency, which is a process that leads to a decrease in placental blood flow and/or transplacental transfer of oxygen and nutrients to the fetus [4,5]. Hence, intrauterine ischemia/hypoperfusion models have probably been the most important model to study the pathophysiology and consequences of LBW-FGR [5]. Most of the studies in models of intrauterine ischemia/hypoperfusion have focused on the effects on the central nervous system [6,7,8]. Other studies have focused on one or two specific organs alone [2,3]. To the best of our knowledge, no such study has assessed the effects systematically, that is, on multiple organs including the brain, muscles, and splanchnic organs. Systematic analysis allows us to better understand which organs are susceptible to intrauterine hypoperfusion and which organs are relatively resistant to it.

Previously, we developed a rat model of LBW due to FGR induced by mild intrauterine hypoperfusion [7,9]. In our model, all fetuses are subjected to a similar degree of persistent mild hypoperfusion through stenosing all four arteries irrigating uterus, i.e., the bilateral ovarian and uterine arteries, with metal microcoils wrapped around them on embryogenic day 17 (E17), which is thought to be equivalent to embryonic week 20 in human fetuses [10]. In most other models, intrauterine hypoperfusion is produced through either completely blocking the blood flow to the uterus by means of clipping all the four arteries and unclipping after 10–60 min (models of transient complete ischemia followed by reperfusion) or partially blocking the blood flow via suture ligation of either the ovarian or uterine artery. In this case there is a gradient of reduction in blood flow to the uterine horn; fetuses located close to the ligation are subjected to severe ischemia, while fetuses located far from the ligation are barely subjected to hypoperfusion (models of persistent hypoperfusion with gradient). We considered our model a useful model to study the consequences of FGR due to placental insufficiency [7,9]. We reported that the model presented behavioral changes and mild morphological brain injuries [7]. Splanchnic organs and skeletal muscles were not assessed in this model. We hypothesized that intrauterine hypoperfusion results in hypotrophy in multiple organs with some inter-organ variability. Studies on splanchnic organs and skeletal muscles in human or rodents with LBW are limited, and the reported results are often inconsistent [11,12,13,14,15]. The present study’s first aim was to determine the influence of LBW-FGR on splanchnic organs and skeletal muscles in this rat model.

The gut microbiota plays a crucial role in healthy development of not only gastrointestinal system but also many other organ systems [16]. LBW infants have altered gut microbiota compared with normal-birth-weight infants [17]. Although bifidobacteria become dominant by three months of age in healthy term infants [18], LBW infants show reduced levels of bifidobacteria [17,19]. Among the bifidobacteria populating the intestines of healthy infants, *Bifidobacterium longum* is the most dominant species, followed by *Bifidobacterium breve* (*B. breve*) and *Bifidobacterium bifidum* [20]. *B. breve* M-16V is a strain commonly used as a probiotic for infants, and its early oral supplementation has been shown to promote the acquisition of beneficial commensal bacteria in preterm LBW neonates [21,22]. Our previous study showed that the gut microbiota in animals with LBW-FGR was different from that in sham control animals during adolescence, and that early oral supplementation of *B. breve* M-16V normalizes such differences [23]. A meta-analysis of non-randomized control trials (RCTs) (not RCTs) showed significant benefits of the supplementation of *B. breve* M-16V on late-onset sepsis and mortality in preterm infants [24]. *B. breve* M-16V is suggested to regulate immune balance and inflammatory response [25]. We hypothesized that early oral administration of *B. breve* M-16V could alleviate the influence of LBW-FGR on splanchnic organs and skeletal muscles, i.e., the probiotic could ameliorate putative hypotrophy through promoting the acquisition of normal gut microbiota and modulating immune/inflammation. The second aim of the present study was to investigate the effects of the oral supplementation of *B. breve* M-16V during the neonatal and infantile periods on splanchnic organs and skeletal muscles in the rat model of LBW-FGR.

## 2. Materials and Methods

### 2.1. Animals and Surgery

Pregnant female Sprague Dawley rats (Japan SLC, Inc., Hamamatsu, Japan) (*n* = 9) were used for this study. Dams were housed in a standard environment on a 12 h light/dark cycle (lights on at 7 a.m.) and provided ad libitum access to water and a regular diet (MF diet, Oriental Yeast Co., Ltd., Tokyo, Japan). On gestational day 17 (G17), six dams were subjected to mild intrauterine hypoperfusion as previously described [7,9]. Briefly, to reduce uterine blood flow via artery stenosis, metal microcoils with inner diameters of 0.24 mm were wrapped around each proximal part of the ovarian and uterine arteries on each uterine horn. Three dams were subjected to anesthesia, but laparotomy was not performed, and their offspring were served as control animals. Our previous study showed no difference in birth weight or body weight gain between the offspring of non-surgery control dams and the offspring of sham-surgery dams with laparotomy [7].

### 2.2. Group Allocation and Oral Supplementation

The dams were allowed to deliver spontaneously, and pups were born on E21 or E22. Only the males were used for this study, and females were used for a different study. Pups with birth weight less than 5.5 g from dams with mild intrauterine hypoperfusion were defined as LBW pups, since we had observed that a birth weight of 5.5 g was the –2 standard deviation level among normal control pups. The litter size was adjusted to 8–10 pups per litter. Six litters from dams with mild intrauterine hypoperfusion were randomly assigned to either the *B. breve*-administered litter (LBW-B.breve group) or vehicle-administered litter (LBW-vehicle group). To prevent microbial transmission among littermates in the same cage, they were assigned to the same group. Three litters from dams without mild intrauterine hypoperfusion were assigned to the sham group.

**Figure 1:**
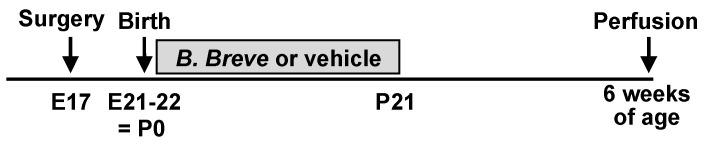
Experimental schedule. Grey box indicates a period of oral supplementation of either *Bifidobacterium breve* (*B. breve*) or vehicle. E: embryonic day; P: postnatal day.

A solution containing *B. breve* M-16V (5 × 10^8^ CFU/mL) was orally administered to the pups in the LBW-B.breve group at 10 μL/g of body weight once daily from the next day of birth, i.e., postnatal day 1 (P1) up to weaning (P21) (Figure 1). Gavage administration of the solution (normal saline) containing *B. breve* and starch as an excipient was performed using a round-tip needle (Natsume Seisakusho Co., Ltd., Tokyo, Japan) inserted into the stomach. The dose of *B. breve* was determined according to previous animal and clinical studies showing its safety and efficacy for other conditions [26,27,28]. *B. breve* M-16V was provided by Morinaga Milk Industry Co., Ltd. (Tokyo, Japan). The LBW-vehicle group and sham group received normal saline containing starch using the same protocol as that used for the LBW-B.breve group. After weaning, the pups were allowed access to water and a regular diet (MF diet; Oriental Yeast Co., Ltd., Tokyo, Japan) ad libitum. All animals, except one in the LBW-B. breve group, survived up to six weeks of age. This rat died on P19, presumably due to the stress of daily needle insertion into the stomach, and not due to the adverse effects of *B. breve*, as some rats had died in our preliminary study with normal saline to test the feasibility of daily gavage administration into the stomach in LBW pups from P1. The animals used in this study were the same as those used in our previous report on behavioral and brain analyses [23].

### 2.3. Analysis of Organs and Their Histology

At six weeks of age, which is considered equivalent to human adolescents, the animals were deeply anesthetized with intraperitoneal administration of butorphanol tartrate, medetomidine hydrochloride, and midazolam. The small and large intestines were removed, and their lengths were measured. The number of Peyer patches was counted macroscopically. The liver, spleen, kidneys, skeletal muscles of the hindlimb, and cecum were removed and weighed. These organs were placed in ice-cold 4% paraformaldehyde in phosphate-buffered saline (PBS). After fixation, the kidneys and small intestines were embedded in paraffin, and thin sections (3 μm thick for the kidney and 4 μm thick for the small intestine) were cut using a microtome. The sections were stained with hematoxylin and eosin (H&E). The number of nephrons in the entire coronal section of the kidney was counted. The number of Paneth cells in 10 randomly selected crypts was counted in the duodenum, jejunum, and ileum. Using ImageJ software version 1.53b (NIH, Bethesda, MD, USA), the heights and depths of 10 randomly selected, intact, well-oriented villi and crypts were measured in the duodenum, jejunum, and ileum. The average number of Paneth cells, height, and depth in each region of the small intestine were used for the statistical analyses. All analyses were performed by investigators who were blinded to the group allocation.

### 2.4. Statistics

Statistical analyses were performed using Prism 8 software (GraphPad Software, San Diego, CA, USA). We always compared the data of three groups: sham, LBW-vehicle, and LBW-B.breve. Temporal changes in body weight were assessed using two-way repeated-measures analysis of variance (ANOVA), followed by Tukey’s test. To assess the number of Peyer’s patches and Paneth cells, the Kruskal–Wallis test, followed by Dunn’s test, was used. For kidney weight, two-way ANOVA followed by Tukey’s test were used. All other analyses were performed using one-way ANOVA, followed by Tukey’s test. The Geisser–Greenhouse correction was used when variances were not equal. The significance level was set at *p* < 0.05. When the *p* value was = or >0.05 but <0.10, the exact *p* value is described in the Results section and shown in the figures. Data are expressed as means ± standard deviation.

## 3. Results

### 3.1. Temporal Changes in Body Weight

**Figure 2:**
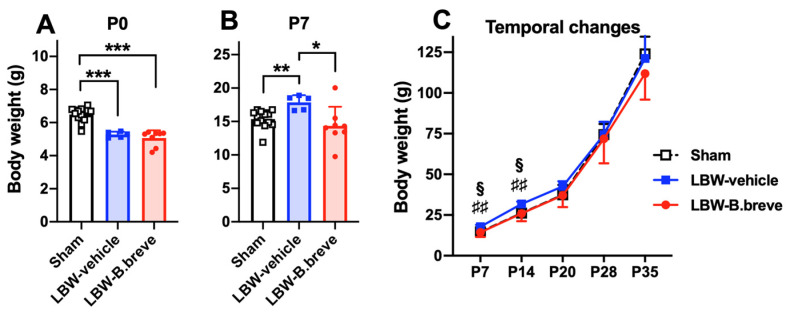
Body weight. Body weight on postnatal day 0 (P0) (**A**) and P7 (**B**). Temporal changes in body weight from P7 to P35 (**C**). Sham, *n* = 13; LBW-vehicle, *n* = 5; LBW-B–B breve, *n* = 7. * *p* < 0.05, ** *p* < 0.01, *** *p* < 0.001, according to one-way ANOVA. ♯♯ *p* < 0.01 Sham vs. LBW-vehicle, § *p* < 0.05 LBW-vehicle vs. LBW-B.breve via repeated-measures two-way ANOVA.

There were three sham-surgery dams; two dams delivered on G21 and one dam delivered on G22. Thirteen male pups were obtained from these. Those 13 pups weighed more than 5.5 g, and they were all allocated to the sham group. Among the six dams with mild intrauterine hypoperfusion, four dams delivered on G21, and two delivered on G22. In total, 13 LBW (defined as less than 5.5 g) male pups were obtained at the time of allocation (P1); a few LBW pups died before allocation. LBW pups were allocated to either the LBW-vehicle (*n* = 5) or LBW-B.breve group (*n* = 8). The mean birth weights of the LBW groups were significantly lower than those of the sham group (Figure 2A). The LBW-vehicle group rapidly increased in weight, and they became heavier than the sham group on P7 (which is considered to be equivalent to the neonatal period in humans) (Figure 2B). However, their body weight became similar to that of the sham group on P20 (roughly equivalent to early childhood in humans), and thereafter up to P35 (equivalent to adolescences in humans) (Figure 2C). In contrast, the body weights of the LBW-B.breve group increased up to that of the sham group on P7 and remained the same as the sham group thereafter (Figure 2B,C).

### 3.2. Solid Organs in the Abdomen

**Figure 3:**
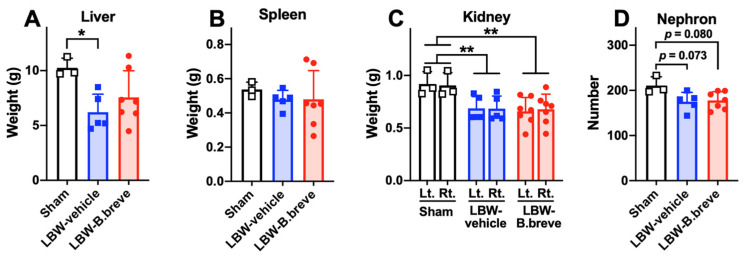
Solid organs in the abdomen. Weight of the liver (**A**), spleen (**B**), and kidneys (**C**) at six weeks of age. The number of nephrons in the entire coronal sections at six weeks of age (**D**). Sham, *n* = 3; LBW-vehicle, *n* = 5; LBW-B.breve, *n* = 7. Left: left; Right: right. * *p* < 0.05, one-way ANOVA. ** *p* < 0.01, via two-way ANOVA.

The liver weight in the LBW-vehicle group, but not in the LBW-B.breve group, was significantly lower than that in the sham group (Figure 3A), although there was no difference in the body weights of the three groups on P35. There was no significant difference in spleen weight among the three groups (Figure 3B). The kidney weights in the LBW groups were significantly lower than those in the sham group (Figure 3C). The number of nephrons in the LBW groups was lower than that in the sham group, but the differences were not statistically significant (*p* = 0.073 for LBW-vehicle and *p* = 0.080 for LBW-B.breve) (Figure 3D). However, if the ratios of organ to body weights are compared among the three experimental groups, the weight differences in the liver or kidney did not reach statistically significant levels, presumably due to the slightly lighter body weights in the LBW groups than in the sham group. Similarly, the number of nephrons per kidney weight was not different among the three groups.

### 3.3. The Small and Large Intestines

**Figure 4:**
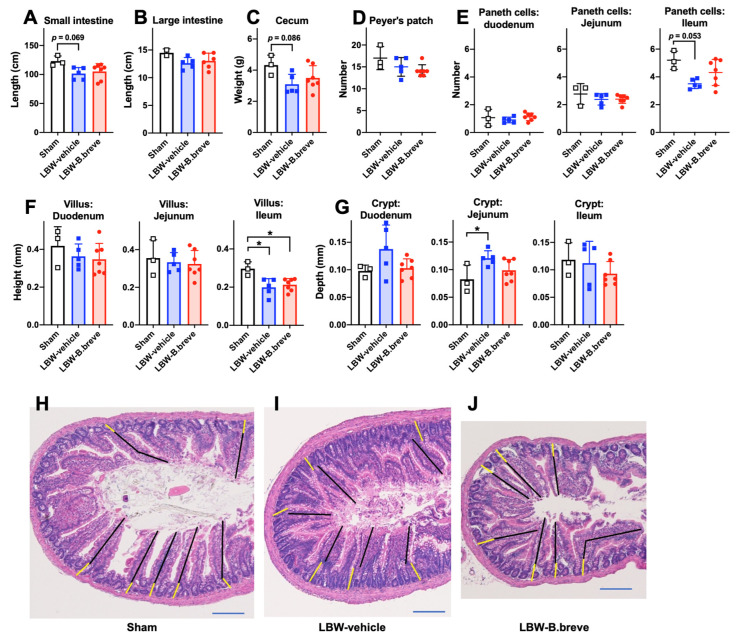
Intestines. Length of the small intestine (**A**) and large intestine (**B**). Weight of the cecum (**C**). The number of Peyer’s patches in the small intestine (**D**) and Paneth cells in 10 randomly selected crypts in the duodenum, jejunum, and ileum (**E**). Average villus heights in the duodenum, jejunum, and ileum (**F**). Average crypt depths in the duodenum, jejunum, and ileum (**G**). All data were obtained at six weeks of age. Sham, *n* = 3; LBW-vehicle, *n* = 5; LBW-B.breve, *n* = 7. * *p* < 0.05, one-way ANOVA. Representative images of hematoxylin and eosin-stained villi and crypts in the jejunum at six weeks of age (**H**–**J**). Scale bar (blue) represents 0.2 mm. Black lines indicate the villus height and yellow lines indicate the crypt depth.

The small intestine in the LBW groups was shorter than that in the sham group, but the differences were not statistically significant (*p* = 0.069 for LBW-vehicle and *p* = 0.12 for LBW-B.breve) (Figure 4A). The length of the large intestine did not differ among the three groups (Figure 4B). The cecum in the LBW-vehicle group was slightly lighter than that in the sham group (*p* = 0.086) (Figure 4C). The number of Peyer’s patches did not differ among the three groups (Figure 4D). Concerning the number of Paneth cells in the small intestine, the LBW-vehicle group had a lower number of Paneth cells in the ileum than the sham group (*p* = 0.053). Concerning villus height in the small intestine, both the LBW-vehicle and LBW-B.breve groups had significantly shorter villi in the ileum than the sham group (Figure 4F,H–J). Concerning the depth of the crypt, only the LBW-vehicle group had altered, i.e., deeper, crypts than the sham group in the jejunum (Figure 4G,H–J). Importantly, when the duodenum, jejunum, and ileum data were analyzed together using two-way ANOVA, the crypt depth in the LBW-vehicle group was slightly deeper than that in the sham group (*p* = 0.095) and significantly deeper than that in the LBW-B.breve group. Apart from the villus height and crypt depth, there was no histologically significant difference among the three groups. The feces characteristics, such as softness, color, odor, and volume, did not differ among the groups, although we did not evaluate them quantitively. Taken together, LBW moderately altered the anatomy of the small intestine, and *B. breve* supplementation partially alleviated these alterations.

### 3.4. The Skeletal Muscles

**Figure 5:**
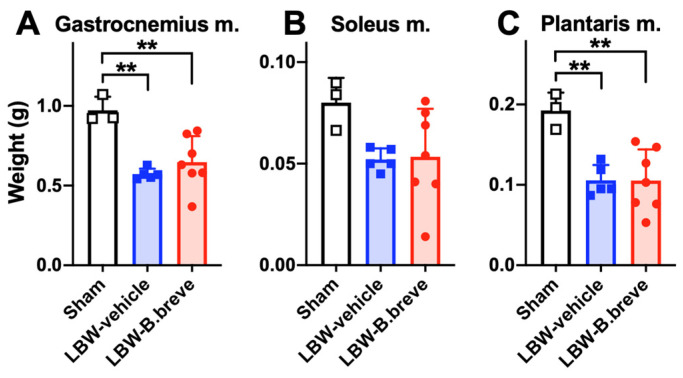
Skeletal muscles. Weight of the gastrocnemius (**A**), soleus (**B**), and plantalis (**C**) muscles at 6 weeks of age. Sham, *n* = 3; LBW-vehicle, *n* = 5; LBW-B.breve, *n* = 7. ** *p* < 0.01, based on one-way ANOVA.

The weights of the gastrocnemius and plantalis muscles in the LBW groups were significantly lower than those in the sham group (Figure 5A,C). There were no significant differences in the weights of the soleus muscle of the LBW groups and the sham group (Figure 5B). When the muscle weights to body weight ratios are compared, the statistical differences among the groups were the same as those compared according to the muscle weights.

## 4. Discussion

The present study showed that rats with LBW due to intrauterine hypoperfusion exhibited alterations in several organs, which may reproduce the conditions associated with health problems seen in people with LBW. Alterations observed in the LBW rats at adolescence were (1) rapid catch-up in body weight during infancy, (2) reduced liver weight, (3) reduced kidney weight, (4) reduced height of villi and increased depth of crypts in the small intestine, and (5) reduced skeletal muscle weight. These changes tended to be milder with *B. breve* M-16V supplementation during the neonatal and infantile periods. Most of the beneficial effects of supplementation were non-significant, but the amelioration of crypt depth in the small intestine was statistically significant.

Individuals with LBW are inherently at risk for developing non-communicable diseases in later life [1,2,29,30]. Among individuals with LBW, those who experienced a rapid increase in weight and/or obesity during childhood are at the greatest risk of non-communicable diseases in later life [31]. In this study, pups in the LBW-vehicle group exhibited rapid catch-up growth and became significantly heavier than the sham pups in early life. *B. breve* supplementation attenuated such overgrowth in LBW pups; this suppressing effect on overgrowth is considered to be beneficial for human infants born with LBW in order to lower the risk of non-communicable diseases in later life.

In situations where intrauterine perfusion is reduced, fetal blood flow is preferentially distributed to the brain to secure the optimal growth of this key organ, thereby reducing distribution to other organs. A necropsy study on human neonates showed that small and large intestinal lengths are reduced in FGR infants compared with appropriately grown infants [11]. The relative weights of the small intestine, spleen, and kidney to the body weight in FGR fetuses of pregnant rats with calorie restriction are significantly lighter than those in control fetuses [32]. These studies in humans and rodents have demonstrated that many splanchnic organs become hypotrophic in the fetal and neonatal periods. Still, a limited number of studies have assessed the status of splanchnic organs in later life, especially in rodent models with intrauterine hypoperfusion. To the best of our knowledge, there is only one study that assessed multiple organ weights in a rodent model with intrauterine hypoperfusion. The liver and kidney in male LBW rats were significantly lighter than in controls in postnatal week 4, while the brain and heart in males were not different from those in controls [33]. These findings are consistent with this study.

Concerning the liver, a study in a rat model with nutritional restriction showed reduced liver weight when assessed up to P21 [12], while another study in a rat model with uterine artery ligation showed no reduction in liver weight when assessed on P14 [13]. A study in a rat model with protein restriction showed hepatic fibrosis and inflammation with no liver weight changes compared with the control assessed at 12 months of age [34]. The influence of LBW on the liver seems to depend on the cause of LBW and timing of the evaluation.

FGR, preterm birth, and LBW are reportedly associated with reduced nephron numbers and consequently higher blood pressure in later life [35]. Human nephrogenesis takes place from weeks 5 to 36 of gestation, and most of them take place during the second half of that period [2]. Rat nephrogenesis begins on E12 and is completed by P8 [2]. A reduction in the nephron number has been shown in several studies, in maternal low-protein-diet models [2,36] and two studies in ischemia/hypoperfusion models [37,38], to the best of our knowledge. Histological alterations in the kidney, that is, dilated diameter and sclerosis of the glomeruli, have been shown in an ischemia/hypoperfusion model [39].

Few studies have assessed the small intestine in rodent LBW models. One such study in a food restriction model showed lower intestinal weight up to 4 weeks but not at 12 weeks of age compared with control rats [14]. A study in a prenatal protein-restriction model showed that none of the parameters evaluated at 35 weeks of age were influenced by birth weight; the parameters were the length of the small intestine, villus–crypt architecture parameters, epithelial cell proliferation and apoptosis, and the activities of intestinal alkaline phosphatase and amino-peptidase [15]. However, when fed a high-fat diet in adulthood, most of the parameters were influenced by birth weight, for example, villus heights in LBW rats were shorter and crypt depths were shallower than those in control rats. The present study showed that the villus height in LBW rats was shorter and crypt depths were deeper than those in control rats. The mechanisms underlying these alterations are unclear, and further studies are warranted to clarify them. To the best of our knowledge, there is no report on the height of villi or depth of crypts in human individuals born with FGR-LBW.

LBW is associated with a higher likelihood of lower lean body mass and sarcopenia in human adults [3,40]. Although the mechanism of the decreased volume of skeletal muscles is hardly known, biopsy studies indicate a trend toward a lower number of myofibers and compensatory myofiber hypertrophy and alteration in fiber-type composition in individuals with LBW [41] and LBW-FGR [42]. As most muscle fibers appear at 18 weeks of gestation in humans [43], the above-mentioned alterations in muscle biopsy are influenced by the timing of adverse intrauterine insult. Primary fibers appear on about E14 and secondary fibers appear between E17 and E21 in rats [43]. Our LBW model rats were subjected to intrauterine hypoperfusion on E17. Hence, it was speculated that the decreased weight of skeletal muscles might have been accompanied with a lower number of muscle fibers. The number and size of myofibers in the gastrocnemius were studied in a similar LBW rat model of intrauterine hypoperfusion; the number was reduced while the cross-sectional area increased, which indicated myofiber loss and hypertrophy [44]. An accelerated aging phenotype and oxidative stress in skeletal muscle have been reported in a rat model with protein restriction [45].

Bifidobacteria are known to exert anti-inflammatory effects and support the intestinal barrier integrity through producing acetate [46,47,48]. *B. breve* induces extensive transcriptome regulation in murine intestinal epithelial cells, including the up-regulation of key genes linked with epithelial barrier function [49]. We performed an analysis of the gut microbiota in the same animal model with the same supplemental protocol at six weeks of age, which was three weeks after the end of the supplementation [23]. There were significant differences in the relative abundances of some genera between the sham group and LBW-vehicle group. No such differences were observed between the sham group and LBW-B.breve group. It was speculated that the beneficial effects of *B. breve* supplementation in this study might have been mediated by its anti-inflammatory and modulatory effects on the gut microbiota. A limitation of this study is that the exact mechanism of action was not explored. The literature shows that *B. breve* M-16V downregulates levels of interleukin (IL)-6, IL-8, and monocyte chemotactic protein (MCP)-1 in porcine intestinal epithelial cells [47]; suppresses IL-1β in colon tissue in a rat model of colitis [50]; reduces the IL-4 level in splenocyte culture; and reduces the serum level of IgE in ovalbumin-sensitized mice [26].

In summary, the rat model of LBW caused by mild intrauterine hypoperfusion reproduced several health problems that human infants with LBW-FGR may frequently suffer in later life; hence, it could be a useful model for studying the mechanisms of LBW-FGR’s harmful effects and for developing therapeutic interventions for these problems. The mechanisms through which LBW-FGR leads to alterations in each organ remain unclear. *B. breve* supplementation in early life may alleviate some of these problems in infants with LBW-FGR. Similarly, the mechanisms underlying the beneficial effects of B. breve supplementation on each organ remain to be clarified.

## Data Availability

The datasets generated and/or analyzed during the current study are available from the corresponding author upon reasonable request.
